# Effect of reducing groundwater on the retardation of redox-sensitive radionuclides

**DOI:** 10.1186/1467-4866-9-12

**Published:** 2008-12-12

**Authors:** QH Hu, M Zavarin, TP Rose

**Affiliations:** 1 Department of Earth and Environmental Sciences, University of Texas at Arlington, Arlington, TX 76019, USA; 2 Lawrence Livermore National Laboratory, 7000 East Avenue, MS L-231, Livermore, CA 94550, USA

## Abstract

Laboratory batch sorption experiments were used to investigate variations in the retardation behavior of redox-sensitive radionuclides. Water-rock compositions were designed to simulate subsurface conditions at the Nevada Test Site (NTS), where a suite of radionuclides were deposited as a result of underground nuclear testing. Experimental redox conditions were controlled by varying the oxygen content inside an enclosed glove box and by adding reductants into the testing solutions.

Under atmospheric (oxidizing) conditions, radionuclide distribution coefficients varied with the mineralogic composition of the sorbent and the water chemistry. Under reducing conditions, distribution coefficients showed marked increases for ^99^Tc (from 1.22 at oxidizing to 378 mL/g at mildly reducing conditions) and ^237^Np (an increase from 4.6 to 930 mL/g) in devitrified tuff, but much smaller variations in alluvium, carbonate rock, and zeolitic tuff. This effect was particularly important for ^99^Tc, which tends to be mobile under oxidizing conditions. A review of the literature suggests that iodine sorption should *decrease *under reducing conditions when I^- ^is the predominant species; this was not consistently observed in batch tests. Overall, sorption of U to alluvium, devitrified tuff, and zeolitic tuff under atmospheric conditions was less than in the glove-box tests. However, the mildly reducing conditions achieved here were not likely to result in substantial U(VI) reduction to U(IV). Sorption of Pu was not affected by the decreasing Eh conditions achieved in this study, as the predominant sorbed Pu species in all conditions was expected to be the low-solubility and strongly sorbing Pu(OH)_4_.

Depending on the aquifer lithology, the occurrence of reducing conditions along a groundwater flowpath could potentially contribute to the retardation of redox-sensitive radionuclides ^99^Tc and ^237^Np, which are commonly identified as long-term dose contributors in the risk assessment in various radionuclide environmental contamination scenarios. The implications for increased sorption of ^99^Tc and ^237^Np to devitrified tuff under reducing conditions are significant as the fractured devitrified tuff serves as important water flow path at the NTS and the horizon for a proposed repository to store high-level nuclear waste at Yucca Mountain.

## Introduction

Major sources of radioactive waste and contamination include the production of nuclear fuels for the weapons program and electricity generation, nuclear weapons tests, fuel reprocessing, and nuclear accidents. In the United States, the total volume of all radioactive waste is 5.5 million m^3^, with a total activity of about 1.2 × 10^9 ^TBq (tera becquerel; 1 TBq = 27.03 Ci) [1]. In addition, there are large volumes of radiologically contaminated soil (30–80 million m^3^) and water (1,800–4,700 million m^3^), especially at U.S. Department of Energy (DOE) facilities that were used for nuclear weapons production [2]. The Nevada Test Site (NTS) is one such DOE facility, with substantial radiologic contamination resulting from nuclear weapons testing and also the location of the proposed Yucca Mountain geological repository for high-level nuclear waste, which has been the focus of radionuclide transport investigations for more than three decades.

Numerous long-lived radionuclides are present in groundwater at the NTS as a result of 828 underground nuclear weapons tests conducted between 1951 and 1992. When weapons testing ended in September 1992, a total of about 4.9 × 10^6 ^TBq of radioactivity was present in the subsurface [3]. Important radionuclides, in terms of abundance, half-life, environmental mobility, and health effects, include ^3^H (tritium), ^14^C (carbon), ^36^Cl (chlorine), ^90^Sr (strontium), ^99^Tc (technetium), ^129^I (iodine), ^137^Cs (cesium), ^237^Np (neptunium), as well as isotopes of uranium (U), plutonium (Pu), and americium (Am). These radionuclides have been commonly identified as the set of contaminants that would cause risk to human health and the environment within the time frame of interest (1,000 years) for the environmental monitoring program at the NTS [4]. Among them, ^3^H, ^14^C, ^36^Cl, ^99^Tc, and ^129^I presumably have large migration potential due to a minimal interaction with the subsurface media.

The objective of the laboratory study described below is to use batch sorption experiments to investigate the impact of groundwater redox conditions on the mobility of selected radionuclides. Thus far, laboratory sorption data acquired for the radionuclide transport at the NTS has been based upon experiments conducted under atmospheric (oxidizing) conditions mainly because of the simplicity of the tests and the considerations that the NTS has primarily oxidizing groundwaters. While this is also true for most of the radionuclide sorption data available in the literature, biotic or abiotic reductive immobilization of radionuclides (U in particular) has gained interest in recent years [e.g. [5,6]]. For this investigation, we conducted a series of batch studies to evaluate the sorption behavior of redox-sensitive radionuclides (Tc, I, U, Np and Pu) under a range of redox conditions spanning those observed in NTS monitoring wells. Some radionuclides that are not redox-sensitive (e.g., Sr and Cs) were also included for comparison. The experiments were conducted using four different types of aquifer materials (alluvium, carbonate rock, devitrified tuff, and zeolitic tuff). Batch sorption experiments were conducted under atmospheric (oxidizing) conditions and in a glove box under five different controlled redox conditions spanning the range from oxidizing to moderately reducing.

## Background

When studying field-scale radionuclide transport, a distribution coefficients (*K*_*d*_) approach has been commonly employed to quantify the extent of radionuclide-aquifer-groundwater interaction. Values of *K*_*d*_, a ratio of the sorbed phase concentration to the solution phase concentration at equilibrium, are used in transport simulations to empirically describe radionuclide/aquifer interactions that are the source of retardation for sorbing radionuclides. The *K*_*d *_data are commonly determined from laboratory-scale batch and column experiments under oxidizing conditions [7] or computed by upscaling mechanistic processes [8]. The term sorption is loosely used in this work to describe the concentration decrease in the solution phase of radionuclides, which could include sorption onto the solid as well as surface precipitation.

Table 1 shows the range in measured *K*_*d *_values for eight radionuclides for representative geologic media, including alluvium, carbonate rock, and volcanic tuffs (devitrified, vitric, and zeolitic) encountered at the NTS [4]. Generally speaking, the largest *K*_*d *_values are observed in the zeolitic tuff and in alluvium, and the smallest values in vitric tuff. Sorption of Pu and Am onto carbonate rock is appreciable, and Np sorption to carbonate rock is higher than other rock types (Table 1).

**Table 1:** Radionuclide sorption on different rock (compiled from [4])

**Element**	**Sample type**	**Number of Samples**	**Distribution Coefficients (mL/g)**			
			
			**Minimum**	**Maximum**	**Mean**	**Standard Deviation**
Strontium	Alluvium	73	1	781	470	197
	Carbonate rock		5	16		
	Devitrified tuff	154	9	1,200	101	141
	Vitric tuff	30	23	220	148	62
	Zeolitic tuff	83	1,200	246,085	39,277	54,882

Technetium	Alluvium	17	0	12	2.16	3.48
	All tuffs		0	0		

Iodine	Alluvium	14	0	24.5	5.74	4.03
	All tuffs		0	0		

Cesium	Alluvium	56	1,720	33,200	5,884	5,153
	Carbonate rock		4	101		
	Devitrified tuff	159	10	3,800	645	656
	Vitric tuff	30	109	1,061	646	317
	Zeolitic tuff	86	2,700	72,000	16,747	13,710

Uranium	Alluvium	48	0.9	60	6.36	5.04
	Carbonate rock		0	132		
	Devitrified tuff	75	0	15	2.51	2.29
	Vitric tuff	59	0	12	1.89	1.7
	Zeolitic tuff	176	0	9,423	45	423

Neptunium	Alluvium	30	1.83	22	8.57	5.08
	Carbonate rock		< 100	5,000		
	Devitrified tuff	421	0	2,353	19	166
	Vitric tuff	400	0	526	3.17	29
	Zeolitic tuff	430	0	22	3	2

Plutonium	Alluvium	24	230	21,000	4,091	4,448
	Carbonate rock		100	10,000		
	Devitrified tuff	118	6	1,900	125	168
	Vitric tuff	71	23	1,810	516	472
	Zeolitic tuff	110	19	2,000	260	242

Americium	Alluvium	24	3,200	1,400,000	174,469	214,582
	Carbonate rock		150	300,000		
	Devitrified tuff	35	79	12,000	1,845	1,834
	Vitric tuff	8	860	2,050	1,354	398
	Zeolitic tuff	25	470	33,000	5,204	7,757

The *K*_*d *_values for Tc and I in Table 1 are based on the assumption that pertechnetate (TcO_4_^-^) and iodide (I^-^) are the dominant species in groundwater and during experimental measurements [7]. However, these and several of the other radionuclides listed in Table 1 (i.e., Tc, I, Np, U, and Pu) are redox-sensitive, and the speciation and retardation of these radionuclides is sensitive to their oxidation state. Variations in groundwater redox conditions, and associated changes in retardation factors are of particular importance to presumably mobile radionuclides, such as ^99^Tc; reducing conditions could result in much longer transport times than those predicted with minimal retardation (i.e., *K*_*d *_close to zero).

### Geochemistry and sorption behavior of redox-sensitive radionuclides

#### Technetium-99

**Figure 1:**
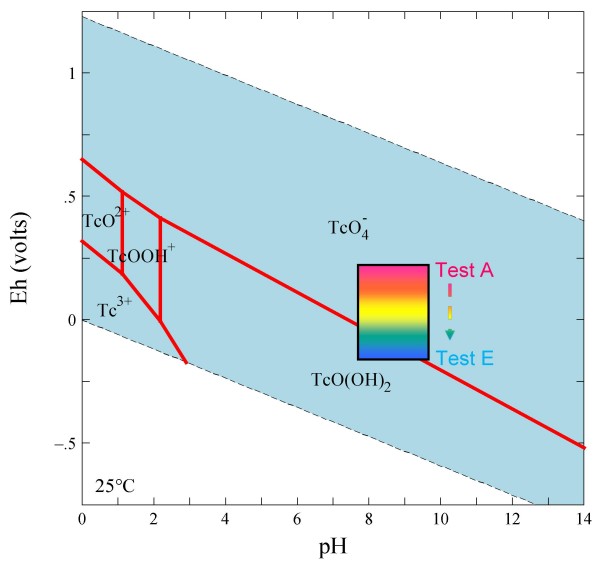
**Eh-pH diagram drawn at 25°C and an equilibrium activity of 10^-11 ^for technetium; stability fields are shown only for the aqueous species**. Diagram was produced using the "thermo" database in the Geochemist's Workbench (version 6.0). Hatched areas are the measured Eh-pH region for Tests A-E conducted in this work.

Technetium exists in valence states ranging from +7 to -1, but in natural environments, the most stable valence states are +7 and +4 under oxidizing and reducing conditions, respectively. Technetium forms a reduced species [predominantly Tc(IV)] at redox potential (Eh) values below about 220 mV with respect to standard hydrogen electrode (SHE) in neutral pH conditions (Figure 1) [9]. At higher Eh, it occurs as Tc(VII)O_4_^-^. Due to its weak interaction with mineral surfaces, TcO_4_^- ^is considered as one of the most mobile radionuclides in the environment. In contrast, lower-valence Tc(IV) species [such as TcO_2_·nH_2_O with n = 1–2; equivalent to TcO(OH)_2_° in Figure 1 when n = 1] are expected to be strongly retarded due to their strong sorption and/or precipitation; the solubility of TcO_2_·nH_2_O(s) in carbonate-containing groundwater was reported to be about 10^-8 ^M [10,11].

The presence of reductants in the host rock (e.g., Fe(II) in pyrite FeS_2_) can contribute to the reduction of Tc(VII) to Tc(IV) [12]. For example, reducing groundwater was observed in boreholes on or near Yucca Mountain, in the western part of the NTS, several of which are known to contain pyrite [13]. Recent work of Hu et al. [14] indicated that the groundwater in the deep NTS aquifer exhibited variable redox conditions. Under reducing conditions, Cui and Eriksen [15,16] reported that TcO_4_^- ^was reduced to TcO_2_·nH_2_O(s) by Fe(II)-bearing fracture-filling minerals on which Tc(IV)_aq _was rapidly sorbed. Reduction of Tc(VII) to Tc(IV) occurred with Fe(II)-containing solid phases but not by aqueous Fe(II) species [11,17-19]. Peretyazhko et al. [19] recently demonstrated that mineral-associated Fe(II) can be an effective heterogeneous reductant of Tc(VII) under anoxic conditions, yielding insoluble Tc(IV) precipitates, coprecipitates, and/or surface complexes that may significantly retard Tc migration. Their experimental results suggested the following affinity series for heterogeneous Tc(VII) reduction by Fe(II): Fe(II) adsorbed on Fe(III) oxides >> structural Fe(II) in phyllosilicates >> adsorbed Fe(II) in phyllosilicates [ion-exchangeable and some edge-complexed Fe(II)] ~aqueous Fe(II).

Lieser and Bauscher [9] observed wide variations in ^99^Tc distribution coefficients for sediment-water experiments performed under aerobic and anaerobic conditions. By varying the redox potential, they observed a change in the *K*_*d *_by about 3 orders of magnitude within a small range of Eh at 190 ± 30 mV for a pH of 7 ± 0.5. Chemical equilibrium modeling using EQ3/6 software has also shown the enhanced retardation of ^99^Tc under reducing conditions in the saturated zone at Yucca Mountain [20]. To summarize, ^99^Tc can behave as either a non-sorbing species (like chloride) or a strongly sorbing species (like Am) – simply because of a modest change in redox conditions. The assumption that ^99^Tc will always behave as a mobile species oversimplifies its geochemical behavior and may overestimate its transport rates.

#### Iodine-129

The fate and transport of ^129^I in groundwater is also dictated by its chemical speciation. Aqueous iodine usually occurs as the highly mobile iodide anion (I^-^). Under more oxidizing conditions, iodine will be present as the iodate anion (IO_3_^-^), which is more reactive than iodide and could be sorbed onto positively-charged sites locally existing in clays and organic matter [21,22]. Unlike other redox-sensitive radionuclides (such as ^99^Tc), iodine sorption may *decrease *under reducing conditions when I^- ^is the predominant species [cf., [23]]. However, coexistence of several iodine species (iodide, iodate, and organic iodine species) has been reported in various aqueous systems, which will tend to make the sorption behavior of iodine more difficult to predict.

#### Actinides

A large volume of literature exists on the geochemical behavior of actinides in the environment; topical review papers include Kim [24], Dozol and Hagemann [25], Silva and Nitsche [26], and Kersting and Reimus [27]. In general, the mobility of actinides in aqueous systems is dependent on (1) their thermodynamic properties, which determine solubility and speciation as a function of pH and redox potential, (2) the availability of inorganic and organic ligands to form soluble complexes, and (3) the composition and abundance of minerals and mineral colloids present in the system. The valence state of redox sensitive radionuclides (including U, Np and Pu) plays a major role in defining the geochemical reactions and migration behavior of these elements. Solubility-limited concentrations, complexation reactions, sorption onto minerals, and colloid formation all differ considerably as a function of oxidation state [28].

**Figure 2:**
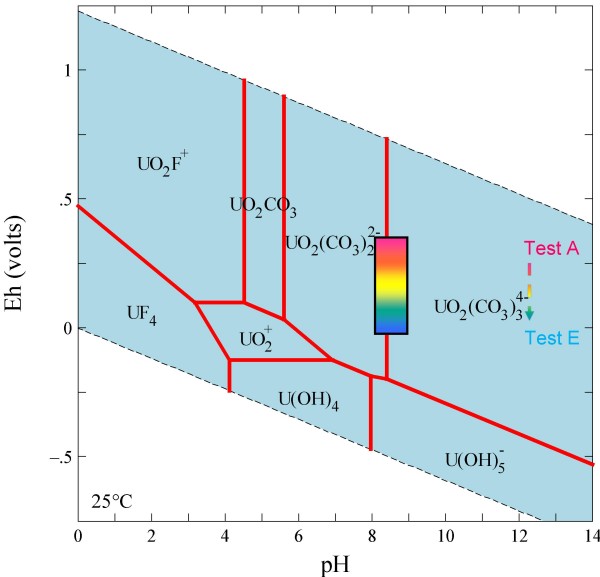
**Eh-pH diagrams drawn at 25°C and a species activity of 10^-13 ^M for uranium in J-13 water**.

The chemistry of uranium in the environment is dominated by the difference in behavior of the U(IV) and U(VI) ions. The tetravalent form generally has low solubility while the hexavalent form is relatively soluble as the uranyl (UO_2_^2+^) ion and its complexes [29]. As shown in Figure 2, the uranyl ion commonly forms soluble complexes with carbonate ligands at pH values typical of NTS groundwaters [30,31]. Even at relatively low oxidation potentials, uranyl species may dominate aqueous uranium speciation, although uraninite (UO_2_) is the stable solid phase. Uranium is more readily sorbed onto minerals or organic matter when present as the positively charged uranyl species, and this step may precede reduction to less soluble U(IV) solids [32-35]. However, the strong affinity of carbonate ligands for uranyl [e.g., [36]] in solution effectively competes with sorption, thereby limiting the sorption of uranyl carbonate complexes [28].

**Figure 3:**
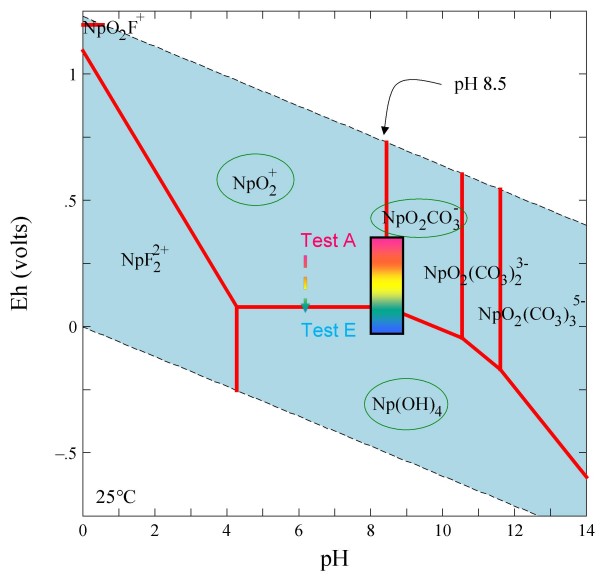
**Eh-pH diagrams drawn at 25°C and a species activity of 10^-13 ^M for neptunium in J-13 water**.

An important feature of neptunium chemistry in aqueous systems is the large stability range for Np(V) [37]. The pentavalent NpO_2_^+ ^species is dominant at pH values < 8 whereas Np(V) carbonate complexes tend to dominate at higher pH values [28] (Figure 3). Since Np(V) solid phases are relatively soluble and Np(V) aqueous species sorb weakly onto common minerals, Np(V) is relatively mobile in the environment. Under reducing conditions, Np(IV) is present as the low solubility Np(OH)_4 _(aq) species at pH values > 5 [28]. Np(IV) shows a strong tendency for sorption to mineral surfaces [37,38], which limits its mobility in aqueous systems.

**Figure 4:**
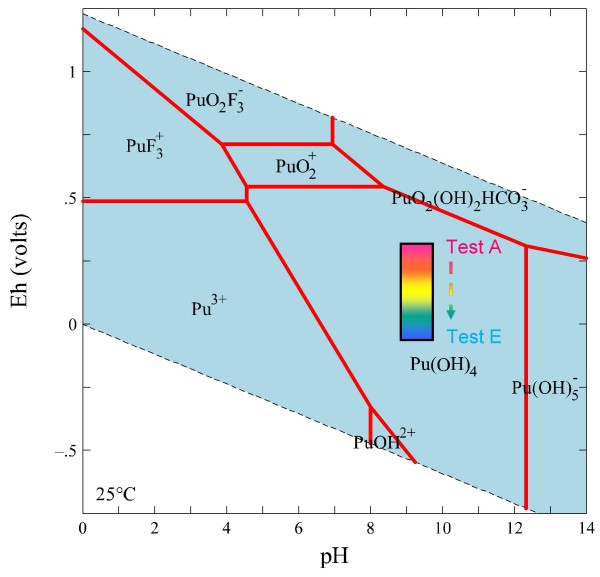
**Eh-pH diagrams drawn at 25°C and a species activity of 10^-13 ^M for plutonium in J-13 water**.

The redox speciation of plutonium is affected by a number of competing variables, and Pu is observed to coexist in multiple valence states in natural waters [39] (Figure 4; note that the stability fields are shown only for the aqueous species). Pu(III) and Pu(IV) tend to be less stable than Pu(V) and Pu(VI) under oxidizing, near-neutral pH conditions, though Pu(IV) exhibits the strongest tendency to form ligand complexes [40]. The aqueous chemistry of plutonium is further complicated by the fact that Pu(IV) disproportionates to Pu(III) and Pu(VI), Pu(V) disproportionates to Pu(IV) and Pu(VI), and Pu(VI) is easily reduced [41,42]. Kersting and Reimus [27] showed that Pu(V) reduction to Pu(IV) is an important mechanism for Pu sorption to mineral surfaces.

**Figure 5:**
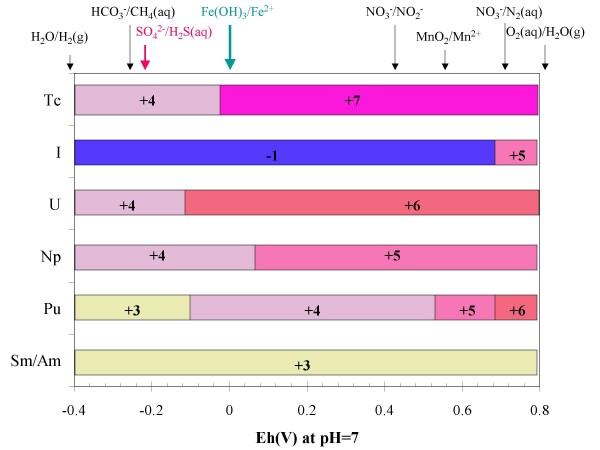
**Expected dominant oxidation states of the radionuclides as a function of standard redox potential under pH 7 in J-13 water at equilibrium with atmospheric CO**_**2**_. Arrows at the top of the figure show the expected redox potentials for common redox couples in the groundwater. This figure was modified from Banaszak et al [43]; results for a pH ~ 9 under which the experiments were conducted in this study will shift the oxidation state boundaries to lower values of Eh.

Figure 5 summarizes the valence states for several radionuclides as a function of redox potentials, and the figure also includes the expected equilibrium redox potentials associated with the common electron-acceptor couples encountered in groundwater [43]. In the laboratory experiments conducted during this study, the redox conditions were controlled by modifying the oxygen concentration in air, and by spiking the solutions with Fe^2+ ^and S^2-^.

### Experimental methods

#### Materials

The extent of radionuclide sorption is dependent on the physical and chemical properties of the radionuclide-aquifer-groundwater system. The lithologic (aquifer) materials used in this study included alluvium, welded/devitrified tuff, zeolitic tuff, and carbonate rock. All lithologic materials were crushed and sieved to a 75–500 μm size range [5]. The alluvium was collected from an exposed section of the U-1a.102 drift in the U-1a tunnel complex (at a depth of 295 m below the land surface) beneath Yucca Flat. The devitrified tuff sample was a Topopah Spring welded volcanic tuff collected from the Yucca Mountain tunnel (~300 m below ground surface). The original glassy matrix in this material has altered to fine-grained crystalline solids that include feldspar, quartz, cristobalite, and some smectite [44]. The zeolitic tuff sample was from drill core UE-7az (496 m below ground surface) within the tuff confining unit (TCU) of Yucca Flat. Zeolitic tuff is formed by the alteration of glassy vitric tuff near and below the water table [45], and is typically comprised of clinoptilolite (> 60%), mordenite, opal, feldspar, and quartz. The carbonate rock was from drill core ER-6-1 (833 m below ground surface) within the lower carbonate aquifer (LCA) at Yucca Flat, and consists of massive dolomite with calcite veinlets.

A companion synthetic groundwater was used for each solid matrix. Synthetic LCA (Ca-Mg-HCO_3_) and TCU (Na-K-HCO_3_) waters were used in carbonate rock and zeolitic tuff sorption experiments, respectively. The solution compositions were based on measured concentrations of major ions in groundwaters sampled from the respective hydrostratigraphic units, except that sulfate, nitrate, and fluoride were omitted from the background solution because of their minimal role in radionuclide retardation processes. Synthetic J-13 water (Na-K-HCO_3 _type) was used for the sorption of radionuclides onto alluvium and devitrified tuff. According to the thermodynamic database "thermo" in the Geochemist's Workbench, a solution composed of J-13 water should be supersaturated with respect to a number of phases, including calcite [46]. To avoid potential mineral precipitation issues, synthetic J-13 water was prepared using a recipe from Viani [46] that is predicted to be stable at 25°C and atmospheric *p*CO_2_. Measured concentrations of constituents in the prepared synthetic background waters are presented in Table 2.

**Table 2:** Chemical composition (mg/L) of background solutions

Constituent	J-13	LCA	TCU
Calcium	3.04	50.33	1.02
Magnesium	1.34	42.6	0.336
Potassium	4.36	22.2	6.608
Silicon	18.6		
Sodium	37.7	109	76.8
Bicarbonate	99.4	447^a^	225
Chloride	4.16	142	14.4
Fluoride	1.13		
Nitrate	3.26		
Sulfate	10.2		
pH (unitless)	7.98	8.08	8.88

^a ^Estimated from the charge balance.

#### Reagents

The radionuclides and surrogate species used in the batch-sorption experiments were chosen because they represent a significant fraction of the radiologic source term at the NTS, and are expected to show a wide range of radionuclide retardation behavior. Synthetic groundwaters were spiked with radionuclides and surrogate species at the appropriate amount to achieve the target concentration (Table 3). The target concentration was based on a combination of instrument detection limits, expected background concentrations, and solubility limits. Tables 3A, 3B, and 3C summarize the measured radionuclide compositions of spiked blank solutions. The radionuclide stock solutions (^99^Tc, ^237^Np, and Pu) were stored in a HNO_3 _matrix. When spiking samples, a small amount of NH_4_OH was added to minimize pH changes to solutions.

**Table 3:** The measured radionuclide compositions of spiked blank solutions

**A. Measured initial radionuclide concentrations (μg/L) in synthetic J-13 water**									
**Test ID**	**Test Condition**	**Sr**	**Tc-99**	**I**	**Cs**	**Re**	**Np-237**	**U**	**Pu-242**

A	atm	3648 ± 117	9.88	12764 ± 1137	1112 ± 38.5	159 ± 5.09	199 ± 5.66	1192 ± 36.2	0.0785 ± 0.004
		(4.16 × 10^-5^)^a^	(9.99 × 10^-8^)	(1.01 × 10^-4^)	(8.37 × 10^-6^)	(8.54 × 10^-7^)	(8.39 × 10^-7^)	(5.01 × 10^-6^)	(3.24 × 10^-10^)
B	1% O_2_	4695 ± 9.62	12.7 ± 0.06	19116 ± 62.2	1469 ± 0.57	209 ± 1.36	261 ± 1.19	1118 ± 34.5	0.113 ± 0.0001
C	0.1% O_2_	4598 ± 11.3	12.4 ± 0.00	18616 ± 679	1439 ± 2.26	204 ± 0.40	258 ± 0.06	1518 ± 11.3	0.111 ± 0.017
D	11 ppm FeCl_2_	3190 ± 162	8.91 ± 0.29	2071 ± 481	993 ± 40.7	151 ± 4.53	148 ± 6.62	393 ± 477	0.032 ± 0.036
E	24 ppm Na_2_S	3134 ± 50.9	8.63 ± 0.12	1090 ± 0.00	986 ± 44.1	159 ± 19.1	162 ± 21.0	656 ± 268	0.030 ± 0.023

Method detection limit		0.1	0.005	20	0.2	0.02	0.002	0.05	0.0005

^a ^Average concentration (mol/L) in parentheses.									

**B. Measured initial radionuclide concentrations (μg/L) in synthetic LCA water**									

**Test ID**	**Test Condition**	**Sr**	**Tc-99**	**I**	**Cs**	**Re**	**Np-237**	**U**	**Pu-242**

A	atm	3630 ± 246	9.56 ± 0.61	11900 ± 990	1109 ± 78.6	159 ± 11.0	200 ± 1.48	1202 ± 91.6	0.100 ± 0.008
B	1% O_2_	4724 ± 76.4	12.4 ± 0.27	18516 ± 84.8	1473 ± 25.5	206 ± 4.41	262 ± 3.56	1587 ± 25.5	0.136 ± 0.001
C	0.1% O_2_	4629 ± 2.26	12.3 ± 0.00	19276 ± 119	1437 ± 9.05	204 ± 0.45	260 ± 0.11	1556 ± 2.83	0.133 ± 0.0002
D	11 ppm FeCl_2_	3656 ± 522	9.72 ± 1.39	1968 ± 87.7	1099 ± 158	162 ± 23.1	139 ± 21.0	62.1 ± 81.0	0.008 ± 0.008
E	24 ppm Na_2_S	3182 ± 83.2	8.53 ± 0.19	846 ± 7.35	966	144 ± 4.36	144 ± 5.32	207 ± 52.1	0.011 ± 0.006

**C. Measured initial radionuclide concentrations (μg/L) in synthetic TCU water**									

**Test ID**	**Test Condition**	**Sr**	**Tc-99**	**I**	**Cs**	**Re**	**Np-237**	**U**	**Pu-242**

A	atm	3786 ± 3.39	9.88 ± 0.02	12252 ± 28.3	1166 ± 7.92	163 ± 0.45	207 ± 0.34	1252 ± 0.57	0.106 ± 0.0004
B	1% O_2_	4720 ± 41.9	12.7 ± 0.06	20788 ± 73.5	1483 ± 11.3	211 ± 1.19	266 ± 3.73	1114 ± 19.2	0.135 ± 0.003
C	0.1% O_2_	4601 ± 0.00	12.1 ± 0.01	18152 ± 67.9	1434 ± 2.26	200 ± 0.40	256 ± 2.66	1549 ± 11.9	0.134 ± 0.002
D	11 ppm FeCl_2_	3149 ± 24.3	8.39 ± 0.04	1896 ± 301	957 ± 10.8	141 ± 0.74	98.6 ± 33.1	962 ± 33.9	0.037 ± 0.042
E	24 ppm Na_2_S	3174 ± 215	8.54 ± 0.57	946 ± 46.4	1018 ± 22.6	180 ± 32.8	190 ± 44.3	1374 ± 314	0.107 ± 0.007

U(VI) was assumed to be the dominant U species in the stock solution based on its stability under oxidizing conditions. The Pu stock solution was purified using a TEVA column. The oxidation state of the Pu stock solution was characterized using both solvent extraction with PMBP (4-benzoyl-3-methyl-1-phenyl-2-pyrozolln-5-one) as described in [47-50] and Pu co-precipitation by lanthanum fluoride [51]. The results indicated that the Pu stock solution consisted of 80% Pu(IV), 15% Pu(III) and 5% colloidal Pu. The ^237^Np stock was purified in concentrated HCl with KI solid using an AG1 × 8 (100–200 mesh) resin column. The Np was eluted from the column using 0.1 M HNO_3_. The Np solution was dried in HNO_3 _on a hotplate before re-dissolving in 1 M HCl to make a Np(V) solution. Np(V) oxidation state of the stock solution was confirmed by UV/VIS spectrometry.

Chemical forms used in the initial solution were Sr^2+^, Cs^+^, I^-^, ^99^TcO_4_^-^, and ReO_4_^-^. Valence states for the actinides were U(VI), ^237^Np(V), and Pu(IV); the predominant chemical forms are expected to be carbonate and/or hydroxide complexes for U, Np, and Pu. These are the valence states and chemical forms expected to occur in the oxidizing groundwaters at the NTS. Cs and Sr are not subject to changes in oxidation state and were included for comparison. ReO_4_^- ^is sometimes used as the surrogate for TcO_4_^- ^based on their similar crystal chemistry, electronic configuration, and thermodynamic data [52-54]. However, there is a difference between Re and Tc in their respective oxidation potentials. A comparison of the E_h_-pH diagrams for Re and Tc shows that ReO_4_^- ^is in equilibrium with ReO_3 _and Re_2_O_3 _over a wide range of conditions. In contrast, there are no equilibrium fields for TcO_3 _or Tc_2_O_3 _[55]. Furthermore, the reduction of Re from +7 to +4 is more difficult than for Tc [55-57].

Radionuclides Pu and ^237^Np used in the batch sorption solutions were from existing stocks at Lawrence Livermore National Laboratory. Standard Reference Materials, SRM 4288A for ^99^Tc, SRM 4341 for ^237^Np, and SRM 4334H for ^242^Pu, obtained from the National Institute of Standards and Technology, were used to prepare the standard solution to calibrate the ICP-MS instrument for measuring the radionuclide concentrations in the batch-sorption samples.

#### Experimental procedure

The first batch test (Test A in Table 3) was carried out under atmospheric conditions. The test was conducted in accordance with ASTM method D4646 [58], except that a solution to solid ratio of 5:1 (one gram of air-dry solid to 5 mL of sorption solution) was employed instead of the 20:1 ratio specified in the ASTM method. All sorption treatments (solid and sorption solution) were run in triplicate. Blank (solid and background solution) and control (no solid; only sorption solution) treatments were run in duplicate.

A 0.7 m^3 ^glove box was used to modify and control the atmospheric composition for the other batch sorption experiments (Tests B-E). Either high purity Ar or a mixture of 99% Ar and 1% CO_2 _was used to control the atmosphere composition inside the glove box. Tests B and C were conducted under a gas composition of about 1 and 0.1% O_2_, controlled by maintaining the flow rate (pressure) of the pure Ar gas. Tests D and E were all conducted at 0.1% O_2 _level, with test tubes further spiked with a reductant (FeCl_2 _for Test D or Na_2_S for Test E) (Table 3). Two gas sensors were placed inside the glove box to monitor air composition. The oxygen sensor (Pro O_2 _analyzer, Nuvair Gas Analyzers, Oxnard, CA) has a detection limit of 0.1% O_2_. A CO_2_/temperature sensor (Model 7001, Telaire, Goleta, CA) was used to monitor the concomitant decrease of CO_2 _with O_2 _and to confirm the decreased O_2 _level inside the glove box. The CO_2 _sensor (working range of ~400 to 1 ppm) is more sensitive than the O_2 _sensor (working range of 21 to 0.1%). The experimental temperature was 22.9 ± 0.5°C.

During the sorption experiments conducted inside the glove box, 15-mL sized centrifuge tubes were uncovered to permit gas exchange and placed on a shaker (Maxi-Mix III type 65800, Thermolyne, Dubuque, IA) at a shaker speed of 1,000 rpm. Batch tests were started when the O_2 _level inside the glove box reached steady-state levels, usually after flushing for 2–4 hours. Then after 48 hours, batch sorption tests were stopped and the sample was filtered with a 0.2 μm PTFE membrane syringe filter (Acrodisc CR 13 mm, Pall Life Sciences, East Hills, NY). The sorption kinetics of radionuclides and the limited mixing during sorption experiments was not evaluated, as this study was intended to compare the effects of redox conditions on sorption under otherwise similar experimental conditions. Furthermore, redox reactions can be kinetically limited, requiring timescales on the order of weeks to achieve steady state [59]. Consequently, observations at 48 hours may not capture the full extent of the redox manipulation. Comparative effects between samples at 48 hours address the initial response of radionuclides to abrupt redox changes.

An aliquot of filtrate (0.5 mL) was pipetted into 3.5 mL 2% HNO_3 _solution for subsequent ICP-MS (Thermo Electron Model X7, Thermo Fisher Scientific, Inc, Waltham, MA) analysis of all elements of interest; 5 ng/L of ^6^Li, ^45^Sc, ^115^In, and ^209^Bi were included as internal standards. The redox potential, relative to the standard hydrogen electrode, was measured using platinum as a sensing electrode and silver-silver chloride as a reference electrode (Thermo Orion Eh combination electrode, model 9678BN, Thermo Fisher Scientific, Inc, Waltham, MA) filled with saturated potassium chloride. Standard Zobell's solution (a solution of potassium ferric-ferrocyanide of known Eh) was used to verify the working operation of the measurement system [60]. Measurement of pH for the filtrate was conducted using an Oakton meter (Eutech Instruments, Singapore) and glass pH electrode system (Orion Ross combination pH electrode, model 81-02) calibrated to three pH buffer standards (pH 4, 7, and 10). Dissolved oxygen in the filtrate was evaluated with a SympHony SB50D (Thermo Orion) with a detection limit of 0.1 mg/L. All these measurements, except for ICP-MS analyses, were performed inside the glove box under the desired O_2 _level.

#### Evaluation of redox conditions

The principal redox couples in the subsurface are O_2_/H_2_O, NO_3_^-^/N_2_, Mn(IV, III)/Mn(II), Fe(III)/Fe(II), SO_4_^2-^/H_2_S, and CO_2_/CH_4_, which form a redox ladder from the most oxidizing O_2_/H_2_O to the reducing CO_2_/CH_4_couples. The theoretical (thermodynamically calculated) Eh values at pH 7 are 816, -182, and -215 mV for the O_2_/H_2_O, Fe(III)/Fe(II), and SO_4_^2-^/H_2_S redox couples employed in this study. However, various authors suggest caution in using Eh to quantify redox condition [61,62]. The difficulty in interpreting redox from Eh measurements results from using an equilibrium approach to describe a highly dynamic system [63]. Eh is a simple measure, but it gives at best only qualitative assessment of water redox conditions because the Pt electrode may not respond to many important redox couples [64]. Thus, a wide range of Eh has been observed for the same redox couple and, as a result, several redox reactions may be relevant within the same Eh range [64]. The measured Eh usually reflects "non-equilibrium" potentials and can only be qualitatively interpreted. Supplementary measurements of redox couple concentrations should be made in addition to Eh measurements to better evaluate redox conditions.

In this work, Eh values measured with a platinum electrode did not appear to reflect the Eh difference between the SO_4_^2-^/H_2_S and Fe(III)/Fe(II) redox couples. In addition to the Eh measurements, sulfate production in the sulfate/sulfide redox couple was monitored. In Test E where 24 mg/L (0.3 mM) Na_2_S was spiked into the sorption solution, about 2 mg/L sulfate (SO_4_^2-^) was produced. According to the reaction:

(1) S^2- ^+ 4H_2_O = SO_4_^2- ^+ 8H^+ ^+ 8 e^- ^

this amount was equivalent to 0.67 mg/L sulfide (S^2-^) being oxidized and suggested that sufficient sulfide remained in solution to influence redox conditions.

## Results and discussion

### Utility of glove box to control redox conditions

**Figure 6:**
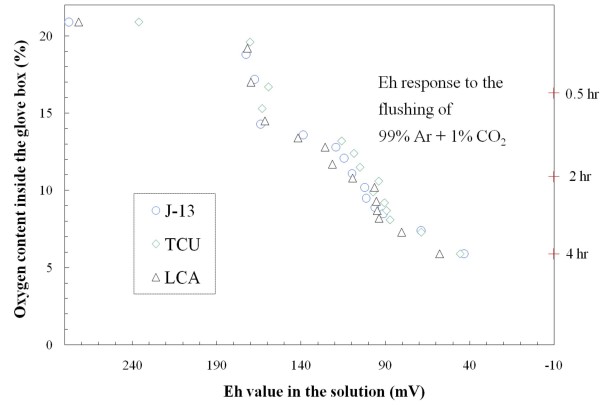
**Change of solution Eh in response to the gas composition (99% Ar and 1% CO_2_) inside the glove box for three different background solutions**.

**Figure 7:**
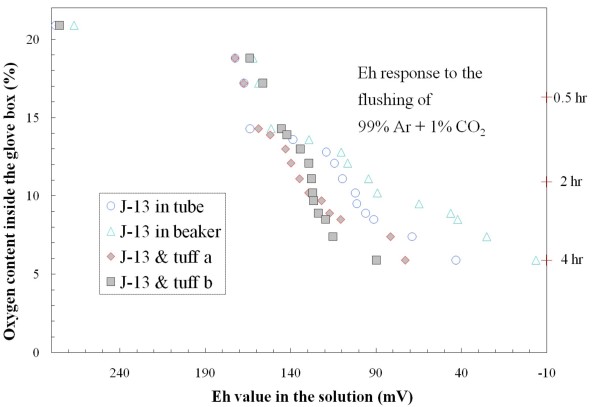
**Change of solution Eh in response to the gas composition (99% Ar and 1% CO_2_) inside the glove box for J-13 water and devitrified tuff (*tuff a *and *tuff b *are duplicate runs inside centrifuge tubes)**.

We first evaluated the approach of producing and maintaining different redox conditions in the radionuclide-aquifer-groundwater system by controlling the air composition inside a glove box. This was accomplished by replacing the ambient air with 1% CO_2 _in Ar. The change of oxygen content as a function of flow rate was controlled by adjusting incoming gas pressure; a steady-state level of O_2 _inside the glove box was reached at about 2 hours under 10 psi and at 4 hours under 1 psi. Figures 6, 7 show the change of solution Eh in response to the flushing-out process of air inside a glove box. The Eh was periodically measured in background solutions, and all three background solutions exhibited a similar trend (i.e., similar behavior in redox buffering) (Figure 6). Figure 7 shows the effect of gas-water interface area and the redox buffering capacity of the crushed rock (devitrified tuff, in this case). As expected, J-13 water placed in a centrifuge tube (1.8 cm^2 ^gas-water interface) responded, in terms of solution Eh, more slowly than that in a beaker with a gas-water interface area 9 times larger. The presence of solids (devitrified tuff, which provides buffering capacity) contributed to the maintenance of a higher solution Eh. Limited measurements of solution pH in J-13 water showed a slight decrease from 7.91 at 20.9% O_2 _to 7.67 at 10.3% O_2_, while pH value for J-13 and devitrified tuff at the same O_2 _levels decreased from 8.0 to 7.2.

The test demonstrates the feasibility of achieving a range of more than 200 mV in Eh under these experimental condition; further reduction can be obtained by applying a reductant (FeCl_2 _or Na_2_S) in the sorption system. For Tests D and E, a small amount of FeCl_2 _or Na_2_S was added into the sorption solution, and pH was adjusted (with either NH_4_OH and/or HNO_3_) to be the same as the value before the spiking of the reductant.

### Sorption in radionuclide-aquifer-groundwater systems

**Figure 8:**
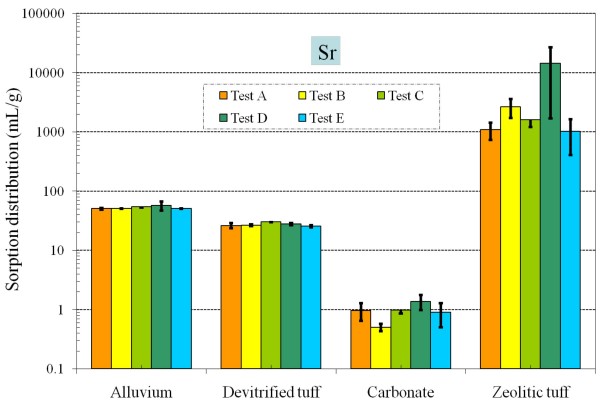
**Change of Sr *K*_*d *_values under five different redox conditions for different aquifer materials**.

**Figure 9:**
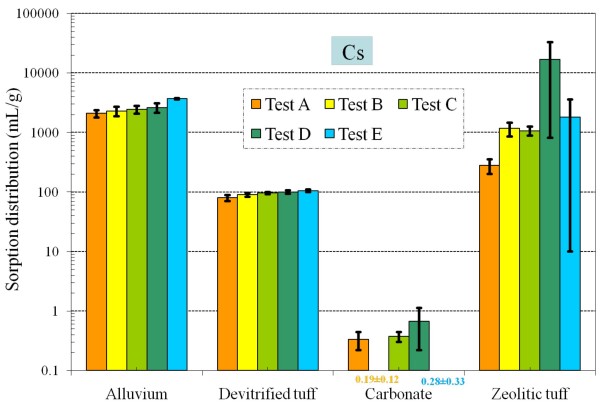
**Change of Cs *K*_*d *_values under five different redox conditions for different aquifer materials**.

**Figure 10:**
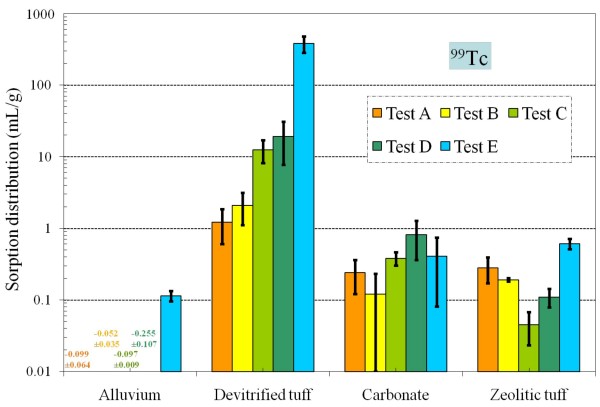
**Change of ^99^Tc *K*_*d *_values under five different redox conditions for different aquifer materials**.

**Figure 11:**
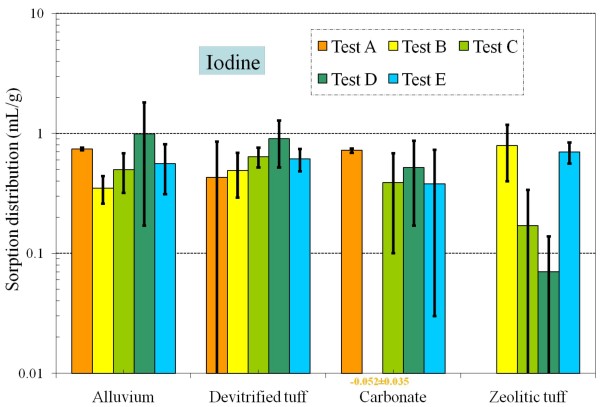
**Change of iodine *K*_*d *_values under five different redox conditions for different aquifer materials**.

**Figure 12:**
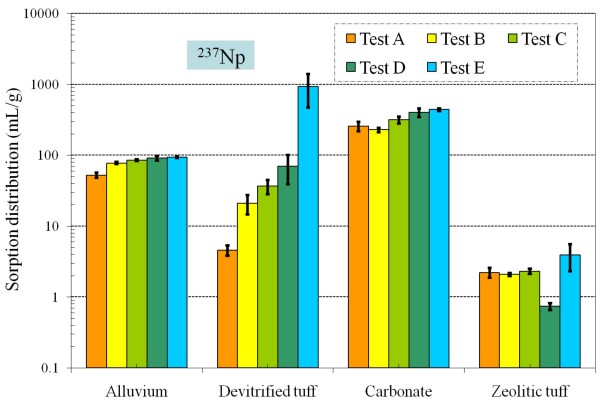
**Change of ^237^Np *K*_*d *_values under five different redox conditions for different aquifer materials**.

**Figure 13:**
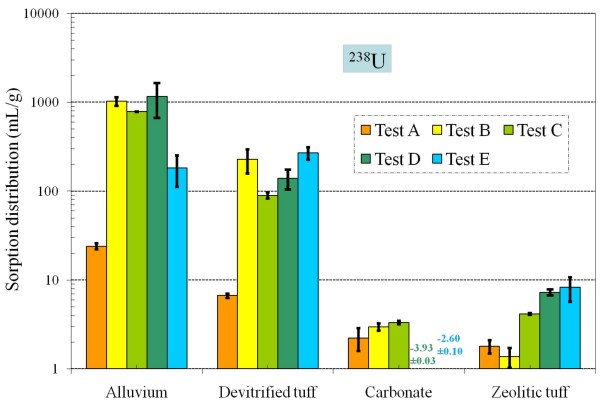
**Change of U *K*_*d *_values under five different redox conditions for different aquifer materials**.

**Figure 14:**
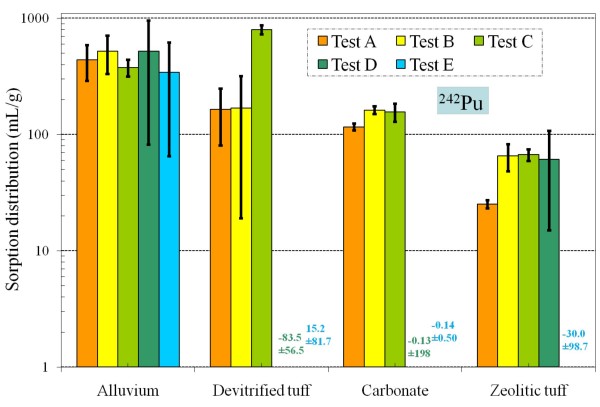
**Change of ^242 ^Pu *K*_*d *_values under five different redox conditions for different aquifer materials**.

Table 4 and Figures 8, 9, 10, 11, 12, 13, 14 present the measured *K*_*d*_s (average ± standard deviation for triplicates) of each radionuclide onto four different aquifers under six different redox conditions. *The measured values were not necessarily the sorption coefficients at equilibration; the objective of these experiments was to study the relative effect of redox condition on radionuclide sorption under otherwise similar conditions*.

**Table 4:** The measured Kds (average ± standard deviation for triplicates) of each radionuclide onto four different aquifers under five different redox conditions

**A. Distribution coefficients (*K*_*d*_, mL/g) for radionuclides sorbed to alluvium in synthetic J-13 water**									
**Test ID**	**Test Condition**	**Sr**	**Tc-99**	**I**	**Cs**	**Re**	**Np-237**	**U**	**Pu-242**

A	atm	50.4 ± 1.65	(-0.099) ± 0.064	0.74 ± 0.02	2078 ± 304	(-0.20) ± 0.07	52.3 ± 4.40	23.9 ± 1.93	437 ± 147
B	1% O_2_	50.6 ± 0.54	(-0.052) ± 0.035	0.35 ± 0.09	2275 ± 415	(-0.03) ± 0.05	77.4 ± 2.48	1022 ± 114	520 ± 188
C	0.1% O_2_	54.5 ± 1.41	(-0.097) ± 0.009	0.50 ± 0.18	2439 ± 363	(-0.28) ± 0.26	85.1 ± 1.83	782 ± 7.06	376 ± 62.5
D	11 ppm FeCl_2_	56.6 ± 9.65	(-0.255) ± 0.107	0.99 ± 0.82	2617 ± 475	0.014 ± 0.44	91.0 ± 7.15	1155 ± 490	518 ± 436
E	24 ppm Na_2_S	50.6 ± 0.52	0.114 ± 0.019	0.56 ± 0.25	3663 ± 92.4	0.44 ± 0.05	93.8 ± 2.83	182 ± 70.4	342 ± 277

**B. Distribution coefficient (*K*_*d*_, mL/g) for radionuclides sorbed to devitrified tuff in synthetic J-13 water**									

**Test ID**	**Test Condition**	**Sr**	**Tc-99**	**I**	**Cs**	**Re**	**Np-237**	**U**	**Pu-242**
A	atm	26.0 ± 2.60	1.22 ± 0.62	0.43 ± 0.83	79.6 ± 9.35	0.20 ± 0.48	4.55 ± 0.75	6.64 ± 0.35	164 ± 83.4
B	1% O_2_	26.5 ± 0.77	2.10 ± 1.00	0.49 ± 0.20	89.6 ± 6.65	0.15 ± 0.029	20.9 ± 6.34	226 ± 68.2	168 ± 149
C	0.1% O_2_	30.2 ± 0.38	12.5 ± 4.41	0.64 ± 0.12	96.1 ± 4.00	0.020 ± 0.025	36.5 ± 8.40	89.4 ± 6.93	798 ± 70.6
D	11 ppm FeCl_2_	27.7 ± 1.16	19.1 ± 11.4	0.90 ± 0.38	99.5 ± 6.40	0.263 ± 0.245	69.6 ± 30.7	139 ± 34.7	(-83.5) ± 56.5
E	24 ppm Na_2_S	25.4 ± 0.92	378 ± 98.0	0.61 ± 0.13	105 ± 6.08	0.56 ± 0.29	930 ± 462	268 ± 42.6	15.2 ± 81.7

**C. Distribution coefficients (*K*_*d*_, mL/g) for radionuclides sorbed to carbonate rock in synthetic LCA water**									

**Test ID**	**Test Condition**	**Sr**	**Tc-99**	**I**	**Cs**	**Re**	**Np-237**	**U**	**Pu-242**
A	atm	0.96 ± 0.32	0.24 ± 0.12	0.72 ± 0.03	0.33 ± 0.11	0.012 ± 0.092	255 ± 39.1	2.22 ± 0.63	116 ± 8.00
B	1% O_2_	0.50 ± 0.07	0.12 ± 0.16	(-0.11) ± 0.01	0.19 ± 0.12	(-0.11) ± 0.05	228 ± 16.1	2.96 ± 0.28	162 ± 12.6
C	0.1% O_2_	0.98 ± 0.12	0.38 ± 0.08	0.39 ± 0.29	0.37 ± 0.07	0.23 ± 0.07	314 ± 36.5	3.31 ± 0.14	156 ± 27.6
D	11 ppm FeCl_2_	1.37 ± 0.39	0.81 ± 0.45	0.52 ± 0.35	0.67 ± 0.45	0.32 ± 0.35	402 ± 55.1	(-3.93) ± 0.03	0.13 ± 198
E	24 ppm Na_2_S	0.89 ± 0.39	0.41 ± 0.33	0.38 ± 0.35	0.28 ± 0.33	(-0.07) ± 0.31	440 ± 18.1	(-2.60) ± 0.10	(-0.14) ± 0.50

**D. Distribution coefficients (*K*_*d*_, mL/g) for radionuclides sorbed to to zeolitic tuff in TCU water**									

**Test ID**	**Test Condition**	**Sr**	**Tc-99**	**I**	**Cs**	**Re**	**Np-237**	**U**	**Pu-242**
A	atm	1080 ± 350	0.28 ± 0.11	(-0.70) ± 0.12	278 ± 76.6	(-0.14) ± 0.21	2.21 ± 0.35	1.79 ± 0.30	25.1 ± 2.04
B	1% O_2_	2637 ± 932	0.19 ± 0.01	0.79 ± 0.39	1164 ± 306	0.049 ± 0.039	2.07 ± 0.10	1.37 ± 0.34	65.3 ± 17.0
C	0.1% O_2_	1587 ± 382	0.045 ± 0.022	0.17 ± 0.20	1065 ± 183	(-0.069) ± 0.029	2.31 ± 0.20	4.12 ± 0.09	66.8 ± 7.61
D	11 ppm FeCl_2_	14264 ± 12586	0.11 ± 0.032	0.07 ± 0.71	16762 ± 15951	(-0.039) ± 0.057	0.74 ± 0.083	7.25 ± 0.56	61.0 ± 46.0
E	24 ppm Na_2_S	1019 ± 612	0.61 ± 0.10	0.70 ± 0.14	1790 ± 2208	0.64 ± 0.88	3.93 ± 1.64	8.21 ± 2.53	30.0 ± 98.7

By modifying the air composition and adding a reductant, we achieved an Eh range of nearly 400 mV from Tests A to E measured at the end of 48 hours experimental duration (Table 5). As expected, for elements that were insensitive to redox conditions (i.e., Sr and Cs), *K*_*d *_values generally did not vary as a function of Eh for all four radionuclide-aquifer-groundwater systems (Table 4 and Figures 8, 9); observed small differences for carbonate and zeolitic tuff were probably related to the sample and analytical variability. Compared to the change in Eh, values of pH among all treatments were relatively stable (Table 6). Under atmospheric (oxidizing) conditions (Test A), measured Sr sorption is moderate in alluvium and devitrified tuff, strong in zeolitic tuff, and weak in carbonate (Figure 8). Cs sorption patterns are similar to those of Sr, being the strongest in alluvium, moderate in devitrified and zeolitic tuff, and very weak in carbonate (Figure 9).

**Table 5:** Measured redox potential Eh (mV), referenced to standard hydrogen electrode, in different treatments

**Test ID**	**Alluvium/J-13**			**Devitrified tuff/J-13**		**Zeolitic tuff/TCU**			**Carbonate/LCA**		
	
	J-13 Control^a^	Blank	Sorption	Blank	Sorption	Control	Blank	Sorption	Control	Blank	Sorption
A	362 ± 13	368 ± 4.8	312 ± 9.9	341 ± 11	338 ± 10	357 ± 6.1	340 ± 5.7	319 ± 5.7	345 ± 4.5	353 ± 1.5	335 ± 4.2
B	207 ± 0.14	205 ± 0.64	206 ± 2.0	201 ± 2.4	203 ± 2.0	186 ± 0.215	191 ± 0.35	187 ± 1.2	195 ± 2.8	204 ± 0.4	195 ± 0.4
C	(-50) ± 1.0	(-27) ± 2.8	(-31) ± 1.1	(-41) ± 0.21	(-22) ± 0.31	29.4 ± 0.64	(-8.3) ± 0.21	(-25) ± 0.36	(-35) ± 0.64	(-34) ± 1.6	(-22) ± 1.4
D	(-55) ± 9.9	(-83) ± 0.07	(-82) ± 1.0	(-82) ± 0.78	(-70) ± 1.4	(-98) ± 1.3	(-102) ± 2.8	(-95) ± 0.60	(-54) ± 2.5	(-90) ± 0.14	(-82) ± 0.67
E	(-50) ± 1.0	(-47) ± 1.8	(-63) ± 6.5	(-56) ± 2.4	(-59) ± 2.7	(-52) ± 0.78	(-69) ± 0.21	(-73) ± 0.38	(-52) ± 0.14	(-59) ± 2.2	(-68) ± 0.85

^a ^The alluvium/J-13 control is also applicable for devitrified tuff/J-13 treatment.

**Table 6:** Measured pH values in different treatments

**Test ID**	**Alluvium/J-13**			**Devitrified tuff/J-13**		**Zeolitic tuff/TCU**			**Carbonate/LCA**		
	
	J-13 Control	Blank	Sorption	Blank	Sorption	Control	Blank	Sorption	Control	Blank	Sorption
A	7.63 ± 0.27	7.89 ± 0.01	7.84 ± 0.04	8.09 ± 0.13	7.89 ± 0.09	8.02 ± 0.06	7.91 ± 0.08	7.89 ± 0.03	8.01 ± 0.08	7.69 ± 0.13	7.84 ± 0.09
B	8.50 ± 0.34	8.76 ± 0.02	8.48 ± 0.03	9.08 ± 0.09	8.32 ± 0.03	8.98 ± 0.02	9.27 ± 0.04	9.01 ± 0.01	8.05 ± 0.04	9.26 ± 0.06	8.59 ± 0.02
C	8.72 ± 0.15	8.08 ± 0.02	8.70 ± 0.06	9.38 ± 0.04	8.66 ± 0.06	8.95 ± 0.20	8.30 ± 0.03	9.04 ± 0.04	9.64 ± 0.03	9.35 ± 0.14	8.89 ± 0.23
D	7.79 ± 0.04	8.97 ± 0.09	8.89 ± 0.03	8.57 ± 0.08	8.51 ± 0.06	9.54 ± 0.01	9.66 ± 0.03	9.63 ± 0.08	9.36 ± 0.10	9.50 ± 0.24	9.15 ± 0.03
E	8.40 ± 0.05	9.11 ± 0.17	9.01 ± 0.02	9.50 ± 0.05	8.75 ± 0.05	9.68 ± 0.01	9.85 ± 0.01	9.72 ± 0.01	8.54 ± 0.11	9.51 ± 0.14	9.28 ± 0.04

For devitrified tuff, as solutions become more reducing from Tests A to E, the Tc *K*_*d *_increases (Figure 10); a slight *K*_*d *_increase in Test E (Na_2_S spiked samples) for alluvium and zeolitic tuff samples was also evident (Tables 4a and 4d). Sorption of Re also seems to increase under more reducing conditions (Test E) in devitrified tuff, alluvium, and zeolitic tuff, but the extent of increase was much smaller (Table 4). This was consistent with the greater stability of oxidized Re compared to Tc (discussed in-*Reagents *section). As reported by Lieser and Bauscher [9], if TcO_4_^- ^is reduced by Fe(II) according to the reactions

(2) 3Fe^2+ ^+ TcO_4_^- ^+8H_2_O = 3Fe(OH)_3 _+ TcO(OH)_2_° + 5H^+^,

hydrolytic adsorption of TcO(OH)_2_° on iron (III) hydroxide or coprecipitation with Fe(III) by formation of hydroxo complexes containing Fe-O-Tc bonds can be expected. Once TcO_4_^- ^has been reduced, precipitation or coprecipitation of Tc_2_S_7 _may occur if H_2_S or S^2- ^ions are present and the solubility product of the sparingly soluble Tc_2_S_7 _has been exceeded. Non-zero Tc *K*_*d *_values were measured in all media except alluvium.

For devitrified tuff, the Tc *K*_*d *_value in Test E was 300 times higher than in Test A. It is not clear why the Eh decrease to a moderately reducing condition only has an effect on ^99^Tc sorption/precipitation in devitrified tuff. We spectulate that a combination of factors contribute to the observed effect, and these factors include changes in the sorbate (redox speciation), the sorbent (surface property alteration), and the water chemistry (such as pH and Fe^2+^/S^2- ^concentrations). It is possible that the devitrified tuff has a trace amount of reducing mineral (e.g., pyrite) that is not measurable by X-ray diffraction. The Fe_2_O_3 _content (wt %) measured by X-ray fluorescence was reported to be 0.96 ± 0.092 for devitrified and 0.84 for zeolitic tuff, respectively [44]. The four aquifer materials had different aqueous iron concentrations after contact with background water, and also exhibited different response to added Fe^2+ ^(Table 7). Both alluvium and devitrified tuff released some iron, but the alluvium seemed to be able to sorb added Fe^2+ ^very effectively. Carbonate rock appeared to have effectively sorbed the added Fe^2+ ^in Test D. Zeolitic tuff could release significant amounts of iron, and the amount released decreases from Tests A to C with an associated decrease in Eh value.

**Table 7:** Measured iron concentrations (μg/L) in 0.2-μm filtrate in different treatments

**Test ID**	**Alluvium/J-13**			**Devitrified tuff/J-13**		**Zeolitic tuff/TCU**			**Carbonate/LCA**		
	
	J-13 Control	Blank	Sorption	Blank	Sorption	Control	Blank	Sorption	Control	Blank	Sorption
A	ND^a^	1038 ± 1520	ND	1414 ± 221	557 ± 435	ND	27,752 ± 5,894	26,328 ± 4,882	ND	ND	106 ± 36
B	ND	703 ± 957	15.0 ± 5.7	98.5 ± 7.2	260 ± 347	ND	5,545 ± 4,139	4,465 ± 3,050	ND	6.94 ± 5.00	12.4 ± 21.6
C	160 ± 118	219 ± 32.7	32.1 ± 20.4	639 ± 634	108 ± 46.3	ND	123	3,539 ± 3,136	ND	16.0 ± 10.2	6.47 ± 11.6
D^b^	891 ± 1193	90.0 ± 60.4	5.65 ± 4.65	230 ± 86	279 ± 149	958 ± 1,371	2,185 ± 1,624	1,307 ± 1,330	187 ± 224	ND	171 ± 254
E	ND	865 ± 489	9.84 ± 13.7	199 ± 74.0	82.5 ± 20.8	ND	4,734 ± 491	5,475 ± 4,227	27.3 ± 17.7	ND	33.5 ± 5.56

^a ^ND: not detected; below the detection limit of about 5 μg/L.

^b^"11 ppm" FeCl_2 _was added into both background and tracer sorption solutions used in Test D. Measured Fe^2+ ^concentrations in the control samples are in the range of 0.2 to 1 ppm, which is consistent with the excepted dissolved Fe^2+ ^concentrations from Geochemist's Workbench; the Fe^2+ ^in solution is controlled by the solubility of FeCO_3 _and/or Fe(OH)_2_.

For the interaction of Tc with alluvium, there was essentially no sorption in Tests A to D (Table 4a). The measurable, though small, sorption of Tc in Test E for alluvium and zeolitic tuff suggested that Tc will be slightly retarded if the radionuclide encounters a reducing zone along its flowpath.

Some level of iodine (applied as iodide) sorption was observed in nearly all samples (*K*_*d *_= 0 - 0.99 mL/g). However, this sorption did not appear to be correlated with redox (Figure 11), therefore, some iodine retardation could help limit its transport in the subsurface at the NTS at any redox condition. Furthermore, the range of *K*_*d *_is substantially lower (and narrower) than the range proposed in Table 1, which is from the literature values confounded by iodine speciation (discussed in *Background*).

**Figure 15:**
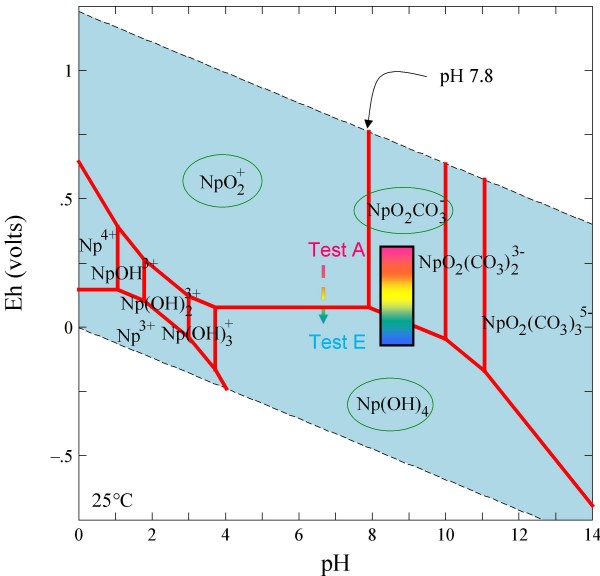
**Stability fields of Np species in Eh-pH diagram for LCA waters**.

Under the atmospheric conditions, Np sorption is the strongest in carbonate rock, moderately strong in alluvium, and moderately weak in devitrified and zeolitic tuff, which is consistent with the literature [4]. Sorption of Np in devitrified tuff increased significantly with decreasing Eh; the Np *K*_*d *_value in Test E was 200 times higher than in Test A. It appears that a substantial quantity of Np(V) was reduced to Np(IV), leading to a strong retention similar to Pu(IV). This suggests that Np may be substantially retarded in mildly reducing groundwater conditions, contrary to most Np transport predictions which assume that Np remains in the +5 oxidation state. Conversely, there was little change in Np sorption on alluvium and carbonate aquifer materials as a function of redox condition, which implies strong Np sorption to minerals in these two materials (e.g., calcite). Similar to what we obtained for Test A under atmospheric condition, low *K*_*d *_values have been reported for Np in tuff [4], while nearly two orders of magnitude larger *K*_*d *_values were reported in carbonate [65]; Figure 12 shows a similar trend. Figure 15 presents the experimental Eh-pH ranges studied in this work overlaid on Np stability field plots for Np in LCA water. The likely Np species encountered in J-13 water would include NpO_2_CO_3_^-^, NpO_2_^+^, and Np(OH)_4 _(Figure 3). The reduced Np(OH)_4 _species is likely to sorb (or precipitate) much more strongly than the oxidized species, which is consistent with our observations of an increasing trend of Np *K*_*d *_with decreasing Eh for devitrified tuff in J-13 water (Figure 3). For Np sorption in carbonate (Figure 15), NpO_2_CO_3_^- ^and NpO_2_^+ ^species might have as strong an interaction with carbonate minerals as Np(OH)_4 _species, which would minimize the effect of Eh (Figure 12).

Overall, uranium *K*_*d *_values in Tests B to E for alluvium, devitrified tuff, and zeolitic tuff (except for Test B) were larger than in Test A, which was conducted under atmospheric conditions (Figure 13 and Table 4C). Measured uranium K_*d*_s to carbonate in Tests D and E were negative (-3.9 and -2.6 mL/g, respectively). Low initial U concentrations in spiked blanks (Table 2b) suggest that precipitation of uranium or losses to container walls had an effect on these samples. The low initial iodine and Pu concentrations in spiked blanks for Tests D and E were also observed (Table 2b). The addition of a reductant consistently affected the initial solution concentrations of U, I, and Pu more than other elements (Table 2); reducing conditions exacerbate issues regarding precipitation or sorption to container walls for these elements.

Using 1 mM sulfide, Beyenal et al. [66] reported the reduction and total removal of about 53,000 μg/L (140 μM) U(VI) from solution. The U(VI) was found to be precipitated as U(IV)-uraninite. We used ~0.33 mM sulfide (24 ppm Na_2_S) and an initial U(VI) concentration of ~1,000 μg/L. The highest *K*_*d *_measured under these conditions was 268 mL/g (98% removal, devitrified tuff); the lowest was 8 mL/g (60% removal, zeolitic tuff). Hua et al. [67] suggested the reaction stoichiometry of U(VI) reduction by hydrogen sulfide could be best described by

(3) HS^- ^+ UO_2_^2+ ^= UO_2 _+ S^0 ^+ H^+^.

The kinetics of U(VI) reduction was found to be largely controlled by the total carbonate concentration and pH. They further concluded that uranium-hydroxyl species, not the dominant U-carbonate species, were the ones being reduced by sulfide. Our experiments suggest that a combination of water chemistry and rock mineralogy will affect the efficiency of sulfide-promoted U(VI) reduction.

Compared to other tests, sorption of Pu is more variable for Tests D and E and most likely the result of Pu loss to container walls, as described earlier. Overall, Pu sorption seems not to be significantly affected by the decreasing redox conditions achieved in this study (Figure 14). This is especially evident for alluvium and zeolitic tuff samples. The predominant Pu species at all redox conditions was the sparingly-soluble and strongly sorbing Pu(OH)_4 _(Figure 4). At low pH (< 7) and highly reducing conditions, Pu may exist as Pu(III). However, this species is also a very strong sorber. Overall, Pu sorption onto NTS aquifers is much less affected by redox than other redox-sensitive radionuclides.

## Conclusion

This work describes the effect of reducing groundwater on the retardation of redox-sensitive radionuclides in the NTS. Based on our review of literature and experimental results, we have the following conclusions:

• Laboratory batch-sorption experiments showed markedly greater retardation for ^99^Tc and ^237^Np in devitrified tuff under reducing conditions. Retardation of radionuclides as a function of redox condition is particularly important for ^99^Tc, which would be highly mobile under oxidizing conditions. The ^99^Tc *K*_*d *_in devitrified tuff correlated with redox, increasing from 1.22 to 378 mL/g from oxidizing to mildly reducing. Under oxidizing conditions, measured ^99^Tc *K*_*d *_values were 0.12–0.81 mL/g in carbonate rock, 0.045–0.61 mL/g in zeolitic tuff, and ~0 mL/g in alluvium.

• Under the experimental conditions, values for *K*_*d *_of iodine are generally not correlated with redox; *K*_*d*_s were 0.33–0.99, 0.43–0.90, 0–0.72 and 0–0.79 mL/g for alluvium, devitrified tuff, carbonate, and zeolitic tuff. Importantly, a non-zero *K*_*d *_was observed in all samples, suggesting that ^129^I transport will be retarded. Including the measurable *K*_*d *_for^129^I iodine would result in less conservative and more realistic radionuclide transport predictions.

• The Np *K*_*d *_strongly correlated with redox condition in devitrified tuff, with an increase from 4.6 to 930 mL/g as experimental conditions became more reducing. The probable reduction of Np(V) to Np(IV), and comparable *K*_*d *_to Pu(IV), under mildly reducing conditions has not been widely reported in the literature.

• The overall trend showed that the sorption of U for alluvium, devitrified tuff, and zeolitic tuff under atmospheric conditions was smaller than the glove-box tests.

• Sorption of Pu was not affected by the decreasing redox conditions achieved in this study, as the predominant Pu species in all conditions was expected to be sparingly-soluble and strongly sorptive Pu(OH)_4_.

The probable presence of reducing groundwater zones in the subsurface could potentially contribute to the retardation of some redox-sensitive radionuclides. Understanding of redox conditions on the transport of redox-sensitive radionuclides garnered from this study at the NTS is very important in assessing potential contaminant mobility and developing remediation strategy in other contaminated sites.

## Competing interests

The authors declare that they have no competing interests.

## Authors' contributions

QHH designed the tests, performed the experiments and speciation modeling, interpreted data, and drafted the manuscript, MZ was involved in data interpretation and revising the manuscript, and TPR assisted with data interpretation and revising the manuscript. All authors have approved the significance of the work, interpretation of results, and contents of the final manuscript.
